# Molecular Determinants of Neuroblastoma

**DOI:** 10.3390/ijms23073751

**Published:** 2022-03-29

**Authors:** Fabio Morandi

**Affiliations:** UOSD Cell Factory, IRCCS Istituto Giannina Gaslini, 16148 Genova, Italy; fabiomorandi@gaslini.org; Tel.: +39-010-5636-3538

The aim of this Special Issue was to discuss novel findings regarding the different mechanisms involved in the progression of neuroblastoma (NB), which represents the most common pediatric extra-cranial solid tumor. Indeed, high-risk NB patients usually display metastatic tumors, with a high frequency of relapse and a survival rate less than 50% [1]. Thus, novel therapeutic strategies are desperately needed, and, in this view, it is crucial to define molecules and pathways that trigger malignant transformation of neuroblasts and metastatic spread, and, possibly, to target them with novel pharmacological compounds in the context of the clinical management of NB patients.

In this view, Foster and coworkers analyzed the effects of pevonedistat, an inhibitor of NEDD8, on NB cell lines in vitro, and in preclinical models of NB [2]. NEDD8 is involved in the neddylation of cullin–RING ligase (CRL) proteins, which target different proteins for ubiquitin-mediated destruction, including those involved in cell cycle progression. Thus, inhibition of the neddylation pathway may induce cell cycle arrest in tumor cells. In this study, the authors demonstrated that pevonedistat induced cytotoxicity in different human NB cell lines, with an IC50 ranging from 136 to 500 nM, independent of p53 status. However, they demonstrated that NB cell lines with wild-type p53 (p53^wt^) undergo cell death through apoptosis, whereas NB cell lines with mutated p53 (p53^mut^) follow a different mechanism. In addition, they demonstrated cell cycle arrest in G0/G1 for p53^wt^ NB cell lines, whereas mutated p53 determined an arrest of the cell cycle in the G2/M phase, with an increased DNA content. Accordingly, CRL proteins are increased in NB cell lines after treatment with pevonedistat, but WEE1, a protein involved in cell cycle progression, was increased in p53^mut^ NB cell lines and decreased in those with p53^wt^. However, preclinical studies performed with orthotopic xenografts of NB demonstrated that pevonedistat is effective in reducing tumor volume, independently of the p53 status of the NB cell lines injected [2]. Thus, this study highlighted a pathway that may be targeted by pharmacological inhibitors in NB, and paved the way for the design of novel phase I/II clinical trials for high-risk NB patients.

Two interesting papers have addressed the role of long non-coding RNAs (lncRNAs), a heterogeneous group of RNA molecules that contribute to various cellular processes in normal and disease states, and are dysregulated in the majority of human cancers, including NB [3,4]. lncRNAs include different types of transcripts, which can be derived from single or multiple exons, introns, antisense strands, enhancers, and pseudogenes. 

Baldini and coworkers summarized, in their review data, the lncRNAs that have been described in NB [3]. The expression of different lncRNAs has been found to be altered in NB. Among them, they reported (i) forkhead box D3 antisense RNA 1 (FOXD3-AS1), which represents an independent prognostic factor of positive outcome in NB, and was found to be downregulated in NB at advanced stages or with poor outcome; (ii) neuroblastoma-associated transcript-1 (NBAT-1) and cancer susceptibility 15 (CASC15), which are located in the same locus and may cooperate as good prognostic factors, being found to be downregulated in high-risk patients; (iii) DLX6 antisense RNA 1 (DLX6-AS1), which is upregulated in advanced stages of NB; (iv) neuroblastoma differentiation marker 29 (NDM29), whose expression is correlated with differentiation and loss of malignity; (v) lncRNA, highly expressed in neuroblastoma 1 (lncNB1) and small nucleolar RNA host gene 1 (SNHG1), whose high expression in NB predicts poor prognosis; (vi) small nucleolar RNA host gene 16 (SNHG16), which increases cell proliferation, migration, and autophagy [3]. In addition, they summarized recent data regarding 20 lncRNAs that are associated with spontaneous regression of NB. Among them, the most interesting were LOC283177 and LOC101928100, which are associated with spontaneous regression and neuronal differentiation [3]. In conclusion, the authors emphasized a possible role of lncRNAs in the prediction of clinical outcome and in the improvement of therapeutic approaches for NB patients [3].

In this view, the study performed by Feriancikova et al. was focused on myocardial infarction-associated transcript (MIAT), which contributes to the development of physiological and pathological processes, including human cancers [4]. In tumors, MIAT acts as a competitive endogenous RNA, plays the role of an miRNA sponge, regulates signaling pathways, and affects some epigenetic modulators. The authors found higher expression of MIAT in *NMYC*-amplified NB cell lines than in those with a single copy of *NMYC*. Furthermore, they found MIAT expression in formalin-fixed paraffin-embedded tissues, with higher MIAT expression in tumor samples from high-risk patients than in those from intermediate-risk patients, and low to absent expression in samples from benign tumors (ganglioneuroma and ganglioneuroblastoma). Since MIAT expression is connected to *NMYC* status, the authors investigated the effects of MIAT silencing, and they found that MIAT-specific siRNA induced apoptosis in *NMYC*-amplified NB cell lines, and inhibited cell growth in NB cell lines with a single copy of *NMYC*. In addition, MIAT silencing down-regulated NYMIC expression in *NMYC*-amplified NB cell lines, and c-myc expression in NB cell lines with a single copy of *NMYC.* Accordingly, silencing of MIAT down-regulated the transcription of both *NMYC* and *c-myc* downstream genes, such as ornithine decarboxylase, in both *NMYC*-amplified and non-amplified NB cell lines [4]. Furthermore, the authors demonstrated that MIAT silencing, and the consequential *NMYC* down-regulation, induced cell cycle arrest of NB cells in the G0/G1 and G2/M phases, with a reduction in cells in the S phase. Accordingly, the proliferation index and migration of NB cells are inhibited by MIAT silencing. Finally, they observed a reduction in glycolytic activity and respiratory capacity in NB cells subjected to MIAT silencing [4]. All these data suggest that MIAT represents a key factor for proliferation, migration, metabolism, and malignant transformation of NB cells, thus representing a potential target for novel therapeutic strategies.

The review by Wulf and coworkers summarized data regarding the role of anaplastic lymphoma kinase (ALK), which may be amplified and/or mutated in NB [5]. Pleiotrophin and midkine are highly expressed in NB, and have been postulated to bind and activate ALK in vitro, leading to increased survival, migration, and differentiation of tumor cells. First, they described different mutated isoforms of ALK, which has been described in NB. Mutations have predominantly been found in the tyrosine kinase motif. In addition, truncated isoforms lacking receptor protein tyrosine phosphatase mu (MAM) and low-density lipoprotein receptor class A (LDL) domains have been described [5]. In addition, the authors summarized data regarding the interaction of ALK with *NMYC* in NB, which are both up-regulated and associated with poor prognosis. They also described the signaling pathways activated by different ALK isoforms, and they summarized data regarding alterations of these pathways during neural crest formation, which may be involved in malignant transformation, and they hypothesized a role of ALK in the migration and proliferation of neural crest cells [5]. On the basis of these observations, they summarized data regarding the treatment of ALK-amplified NB, which are currently based on inhibitors of ALK downstream signaling, rather than ALK inhibitors [5].

Olivera and coworkers addressed the interesting field of pharmacogenetics (PGx), in the context of NB [6]. PGx, which belongs to Precision Personalized Medicine, analyzes the correlations between variants in the constitutive germline DNA of patients, and the efficacy and safety of drugs. The most abundant genetic variants related to PGx are single nucleotide polymorphisms (SNPs), but also genomic insertions, deletions, and repeats, as well as genetic copy number variations (CNVs) [6]. The authors summarized the data available on genetic variants, which can be implemented in clinical settings and on those which require additional studies. The level of scientific evidences needed to discriminate between these two groups is established by drug regulatory agencies, such as Pharmacogenomics Knowledge Base and relevant international consortia of experts who are developing clinical guidelines for PGx implementation [6]. Some drugs currently used in clinical settings for NB patients (i.e., carboplatin, cisplatin, cyclophosphamide, doxorubicin, etoposide, and vincristine) already have clinically valuable PGx recommendations for certain SNPs in different genes, which are correlated to efficacy and toxicity. Other SNPs are currently under investigation for different drugs [6]. The authors finally discussed future perspectives for translational research in the field of PGx, which may include epigenomics and metabolomics [6]. This review is of particular interest, since it demonstrates how the analysis of novel molecular determinants in NB may help to predict the success of therapeutic strategies for high-risk NB patients.

We contributed to this Special Issue by summarizing and discussing the role of tumor-derived extracellular vesicles (EVs) in NB [7]. EVs are sub-micronic vesicles released by cells, either in physiological or pathological conditions, and include different subtypes, which differ in size, morphology, composition, and biogenesis. In this review, we summarized the data present in the literature regarding the possible contribution of EVs in the progression of NB [7]. Different miRNAs have been described in tumor-derived EVs, and some of them are related to malignant transformation and metastatic spread. In addition, a proteomic analysis of NB-derived EVs revealed the presence of tumor-associated antigens, immunosuppressive molecules, and proteins that are involved in cell survival and proliferation, as well as in cancer progression [7]. We summarized data regarding the cross-talk between NB cells and other cell subsets, based on the release of EVs. In this view, mesenchymal stem cells (MSC) acquire a pro-tumorigenic phenotype upon the uptake of NB-derived EVs. NB cells themselves are able to uptake EVs from other NB cells, and such a feature may render tumor cells more aggressive (i.e., cross-talk between Nmyc^+^ and Nmyc^−^ NB cells). Furthermore, NB cells may transfer EVs to immune cell populations (i.e., NK cells and monocytes), or may acquire EVs from them, and the effects of this bi-directional cross-talk may lead to increased or decreased tumor aggressiveness [7]. 

In conclusion, the main topics of this Special Issue, which are summarized in Figure 1, addressed the roles of different molecules, moieties, and genetic variants in the development and progression of NB, and their possible impact on the clinical outcome of patients and on their response to standard treatments.

## Figures and Tables

**Figure 1:**
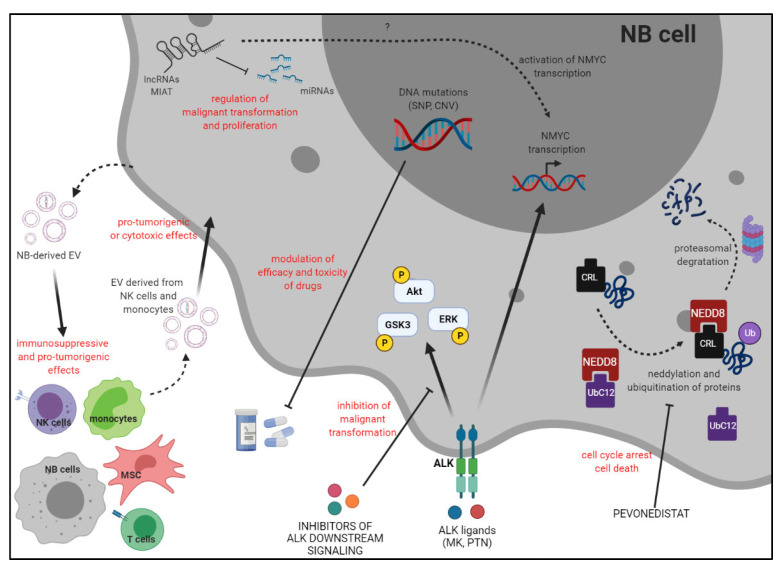
Summary of the main topics addressed in this Special Issue.
